# Estimation of cotton canopy parameters based on unmanned aerial vehicle (UAV) oblique photography

**DOI:** 10.1186/s13007-022-00966-z

**Published:** 2022-12-08

**Authors:** Jinyong Wu, Sheng Wen, Yubin Lan, Xuanchun Yin, Jiantao Zhang, Yufeng Ge

**Affiliations:** 1 Engineering College, South China Agricultural University, Guangzhou, China; 2 National Center for International Collaboration Research on Precision Agriculture Aviation Pesticides Praying Technology, South China Agricultural University, Guangzhou, China; 3 College of Electronic Engineering, South China Agricultural University, Guangzhou, China; 4 College of Mathematics and Informatics, South China Agricultural University, Guangzhou, China; 5 Department of Biological Systems Engineering, University of Nebraska-Lincoln, Lincoln, USA

**Keywords:** Crop height, Leaf area index, Plant phenotyping, UAV, Structure from motion

## Abstract

**Background:**

The technology of cotton defoliation is essential for mechanical cotton harvesting. Agricultural unmanned aerial vehicle (UAV) spraying has the advantages of low cost, high efficiency and no mechanical damage to cotton and has been favored and widely used by cotton planters in China. However, there are also some problems of low cotton defoliation rates and high impurity rates caused by unclear spraying amounts of cotton defoliants. The chemical rate recommendation and application should be based upon crop canopy volume rather than on land area. Plant height and leaf area index (LAI) is directly connected to plant canopy structure. Accurate dynamic monitoring of plant height and LAI provides important information for evaluating cotton growth and production. The traditional method to obtain plant height and LAI was s a time-consuming and labor-intensive task. It is very difficult and unrealistic to use the traditional measurement method to make the temporal and spatial variation map of plant height and LAI of large cotton fields. With the application of UAV in agriculture, remote sensing by UAV is currently regarded as an effective technology for monitoring and estimating plant height and LAI.

**Results:**

In this paper, we used UAV RGB photos to build dense point clouds to estimate cotton plant height and LAI following cotton defoliant spraying. The results indicate that the proposed method was able to dynamically monitor the changes in the LAI of cotton at different times. At 3 days after defoliant spraying, the correlation between the plant height estimated based on the constructed dense point cloud and the measured plant height was strong, with $R^2$ and RMSE values of 0.962 and 0.913, respectively. At 10 days after defoliant spraying, the correlation became weaker over time, with $R^2$ and RMSE values of 0.018 and 0.027, respectively. Comparing the actual manually measured LAI with the estimated LAI based on the dense point cloud, the $R^2$ and RMSE were 0.872 and 0.814 and 0.132 and 0.173 at 3 and 10 days after defoliant spraying, respectively.

**Conclusions:**

Dense point cloud construction based on UAV remote sensing is a potential alternative to plant height and LAI estimation. The accuracy of LAI estimation can be improved by considering both plant height and planting density.

**Supplementary Information:**

The online version contains supplementary material available at 10.1186/s13007-022-00966-z.

## Background

Cotton is one of the most labor-intensive crops; in addition to sowing, pest control and harvesting production links need to rely on a large amount of labor consumption [1]. Due to the acceleration of China’s economic development and urbanization, labor costs are rising rapidly. Currently, China’s cotton planting and labor costs account for more than half of the total cost, approximately 50.9$\%$, and the unit area labor cost was approximately 69.3$\%$ higher than that of the United States [2].

Mechanized cotton harvesting is a significant approach to minimize labor intensity and cotton planting cost, among which cotton defoliation and ripening are important prerequisites and key links to realize mechanical cotton harvesting [3]. Because traditional ground machinery spraying cotton defoliants easily causes damage to cotton fields and boll loss [see Additional file 1(a)], agricultural unmanned aerial vehicle (UAV) spraying has the advantages of low cost, high efficiency and no damage to cotton [see Additional file 1(b)] and has become one of the important defoliant application devices in China’s cotton growing areas [4].

With the rise of new spray equipment in recent years, plant protection UAVs that spray cotton defoliants also lack specific operation procedures [5]. The application volume of plant protection UAVs mainly relies on the operator’s experience for selection, which is generally 15-30 L/ha [6], and it is often difficult to achieve on-demand spraying. The defoliation effect of cotton is not satisfactory when the spraying volume is too small. The higher concentration of the liquid can lead to scorched leaves and hanging branches in cotton [see Additional file 1(c)]. 2. The amount of chemical defoliation used by agricultural UAVs should be calculated based on cotton canopy volume rather than land area [7]. To ensure appropriate defoliation application, the analysis based on the distribution of plant height and LAI of cotton in the field should be performed.

The leaf area index (LAI) is a biophysical parameter in crop phenotypes that refers to the total area of vegetation components (stems, leaves, flowers, fruits, and so on) per unit of land surface area. It is closely related to how plants use light energy and is an important biophysical parameter in crop phenotypes [8, 9]. The LAI is affected by factors such as crop varieties, growing age, nutrient conditions and plant spacing [10]. In precision agriculture, LAI is closely related to plant canopy structure, which is a useful index for crop growth diagnosis, biomass estimation and yield prediction [11, 12]. Timely monitoring of the change in the LAI of cotton after spraying Cotton Defoliant plays an important role in the study of cotton plant defoliation, bolting and yield prediction. Obtaining detailed LAI distribution maps of cotton fields quickly and accurately is very valuable to farmers. These maps can be used by farmers to determine crop growth status based on the existing spatial and temporal LAI information to optimize subsequent crop management decisions [13]. Therefore, accurate assessment of the LAI is crucial in cotton planting management, even if it is a time-consuming and labor-intensive process.

The traditional measurement method for LAI is not only time-consuming and laborious but also easily affects the accuracy of data due to measurement errors [14, 15]. In addition, it easily causes artificial damage to crop plants and affects the normal growth of crops [16]. How to estimate LAI rapidly and nondestructively has become a popular research direction of many researchers [17]. In recent decades, the improvement of unmanned aerial vehicle technology and its application in remote sensing have made remote sensing technology a promising nondestructive technology [18–21]. Remote sensing has been shown to have great potential in estimating LAI for crops and has been applied to rice [22], wheat [23], maize [24] and cotton [25].

Modern technologies based on near-end remote sensing for LAI estimation, such as digital cameras [26] or RGB fisheye lens cameras [27], which can ensure the reliability and speed of LAI estimation while also realizing the estimation of LAI for individual communities. In this case, remote sensing is an advantageous technique that can be used to quickly estimate the LAI of crops, for example, by airborne hyperspectral or multispectral cameras. Different from satellite remote sensing, near-end remote sensing can quickly produce reliable and accurate farmland information maps according to the actual crop growth status and different spectral reflectance of crops [28]. UAV remote sensing uses small aircraft to obtain remote sensing information. Due to its functional diversity and adaptability to user needs, field forms and applications, UAV remote sensing is widely used in precision agriculture applications, providing high-resolution images with space and time. Comba et al. [29] proposed an unsupervised algorithm for vineyard detection and grape row feature evaluation based on 3D point cloud processing generated by UAV multispectral images, which achieved good results in the automatic detection of vineyards, as well as the evaluation of grape row direction and row spacing. Tao Huilin et al. [30] extracted the spectrum based on the UAV hyperspectral image and partial least squares regression method, constructed the estimation model of plant height and LAI of winter wheat, and proved the reliability of the estimation of plant height and LAI. However, the widespread use of professional spectroscopic cameras is limited due to their high cost and complicated data processing procedures [31, 32].

With the evolution of realistic 3D crop model creation technologies, a new method for crop phenotyping research has emerged. As a large point cloud dataset, the 3D crop model contains higher phenotypic parameters of crop plants, which can be obtained directly by lidar scanning or from multispectral and visible images by photogrammetry and computer vision methods [13, 33]. In recent years, a low-cost consumer UAV system composed of light UAVs and RGB cameras has received extensive attention in crop growth monitoring due to its low cost and ease of operation, which has shown high potential in practical applications [34]. The RGB image of the crop canopy obtained by the UAV was used to extract the digital surface model (DSM) by generating point clouds through the Structure from Motion (SfM) method, which can estimate the LAI and other growth indicators [35, 36]. Therefore, there is great interest in using this low-cost method to predict plant height and LAI. A number of studies have confirmed the reliability and effectiveness of evaluating crop phenotypes from dense 3D point clouds. Ivanov et al. [37] were the first to estimate the leaf area of maize using the SfM method, using stereo equipment and having to manually segment the leaves in the image. Mathews [38] created a 3D point cloud model using the SfM method. A simple point cloud processing method was proposed to estimate the LAI distribution of large vineyards. However, the estimated results were susceptible to the influence of low-density point clouds.

Han et al. [39] used the UAV to acquire time-series images for 3D reconstruction of field breeding plots and found that the height of plants determined by the UAV platform was closely related to the height measured manually. For the first time, fuzzy C-means clustering and the set intersection operation were used to analyze the time profile, and multitime sequence analysis of crop field shape was realized. Fawcett et al. [40] made an SfM point cloud model with images in an oil palm forest and used local height maxima to evaluate automatic canopy identification with better canopy segmentation results. It is quite significant to improve the existing methods to further raise the efficiency and practical application of crop parameter acquisition.

Zermas et al. [41] found that the 3D maize model reconstructed based on the SfM method can automatically extract the phenotypic characteristics of a single plant with high precision. Zermas et al. realized that the LAI was estimated with 92.5$\%$ accuracy and that the height of a single corn plant was estimated with 89.2$\%$ accuracy. Previous studies have confirmed the accuracy and validity of estimating crop phenotypic parameters from point clouds obtained by using the SfM method [42, 43], which creates the possibility of achieving low-cost prediction of plant height and LAI for large area crops [44]. However, the method used still has limitations in the extended application, and the generalization ability needs to be improved. For example, due to the complexity of field environments and crop canopies and the characteristics of imaging systems, images containing phenotypic information can vary greatly in terms of resolution, imaging quality, and detail richness, which also leads to obstacles in scaling up existing methods. Therefore, the point cloud model obtained by the SfM method to retrieve the structural parameters of field cotton crops has great potential in precision agriculture.

The overall goal of this work was to evaluate a low-cost UAV method for the rapid acquisition of phenotypic information and 3D point cloud analysis of cotton to monitor cotton plant height and LAI under field conditions. To enrich the point cloud information, the RGB image was obtained by simulating a five-way lens by performing multiple flight missions and then processing the 3D dense point cloud of cotton under field conditions obtained by the SfM method. Based on the dense point cloud. The specific aim of this work is as follows:

1. Combining DSM and manual sampling methods for measuring plant heights to derive ground elevations within the sampled plots. The digital ground elevation map of the test plot was obtained indirectly, and the cotton plant height of nonsampling points was estimated by using the digital ground elevation map.
2. A 3D point cloud of cotton in the field was rapidly constructed from UAV images, which can be used to extract the physical parameters of the crop canopy. A multivariate linear model was used to describe and model the relationship between the defined crop canopy and LAI, which was used to estimate LAI. The LAI obtained with the LAI instrument was compared to verify the reliability and accuracy of the estimated LAI.
3. A graph of LAI over time (10 days after spraying cotton defoliant) was drawn to explore the relationship between plant height as well as LAI and the effect of cotton defoliant spraying.

## Methods

### Experimental site

**Fig. 1:**
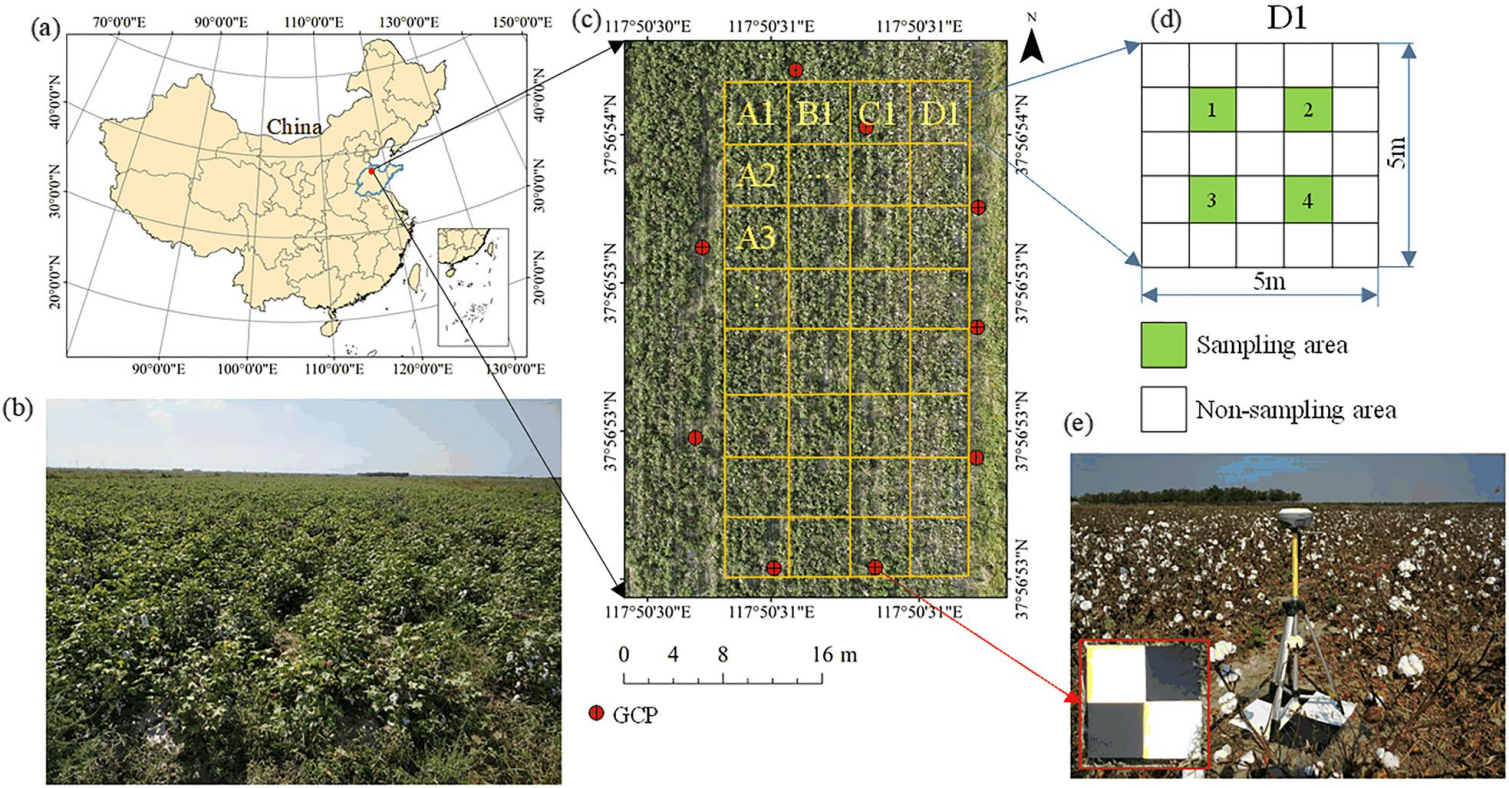
**a** Location of the research site. **b** Experimental cotton field after defoliant spraying. **c**, **d** Location distribution of LAI measurement points. **e** Coordinate information measurement of ground markers

The experiment was conducted at a cotton breeding base from September 27 to October 7, 2020, in Wudi County, Binzhou City, Shandong Province, China. The experimental site is at latitude 37.948182$^\circ$N, longitude 117.841890$^\circ$E, with an average altitude of 4 meters, as shown in Fig. 1a, c. Figure 1c also shows the distribution of ground control points and sampling areas. This area is a traditional cotton growing area with a temperate monsoon continental climate. The size of the experimental area was 315 meters $\times$ 46 meters. Considering the limited endurance of the UAV, the final selected test area was 40 meters $\times$ 20 meters, and weeds in the test area were removed. The cotton variety of the test plot was Lu 54, planted in late April 2020, with a planting row spacing of 60 cm. When the experiment was conducted, the cotton was in the early stage of bouncing, and the average plant height was 60 cm.

### Measurement of field data

**Table 1:** Flight parameters and spraying parameters of UAV

Name	Parameter
Model of UAV	P30 2018
Flying height	1.5 m
Flying speed	3 m/s
Volume median diameter (VMD)	110 µm
Type and dosage of defoliant	Xinthali (50% phenylene $\cdot$ ethylene suspending agent) 270 mL/ha
Type and dosage of synergist	Fatty alcohol polyoxyethylene ether sulfonat 180 mL/ha
Spraying amount of mixed liquid	18 L/ha

**Fig. 2:**
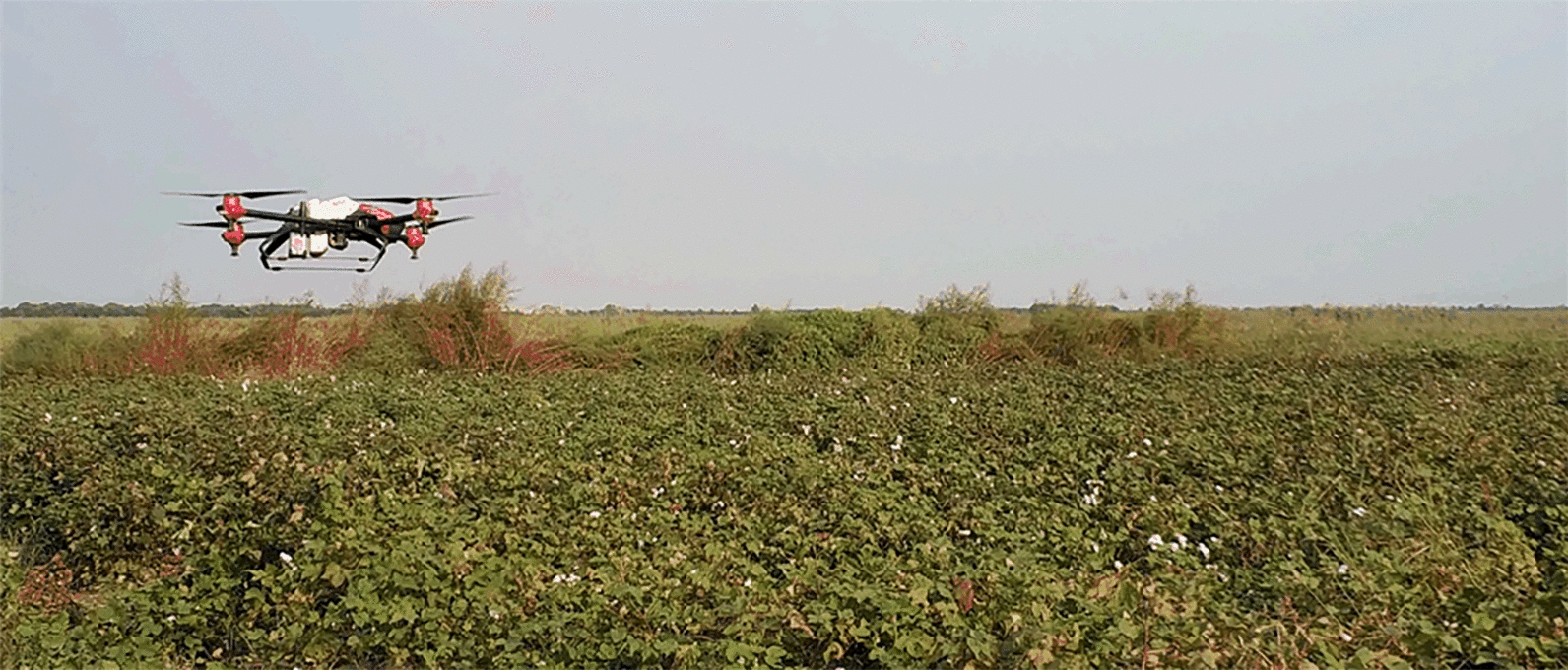
The spraying cotton defoliant operation process

**Fig. 3:**
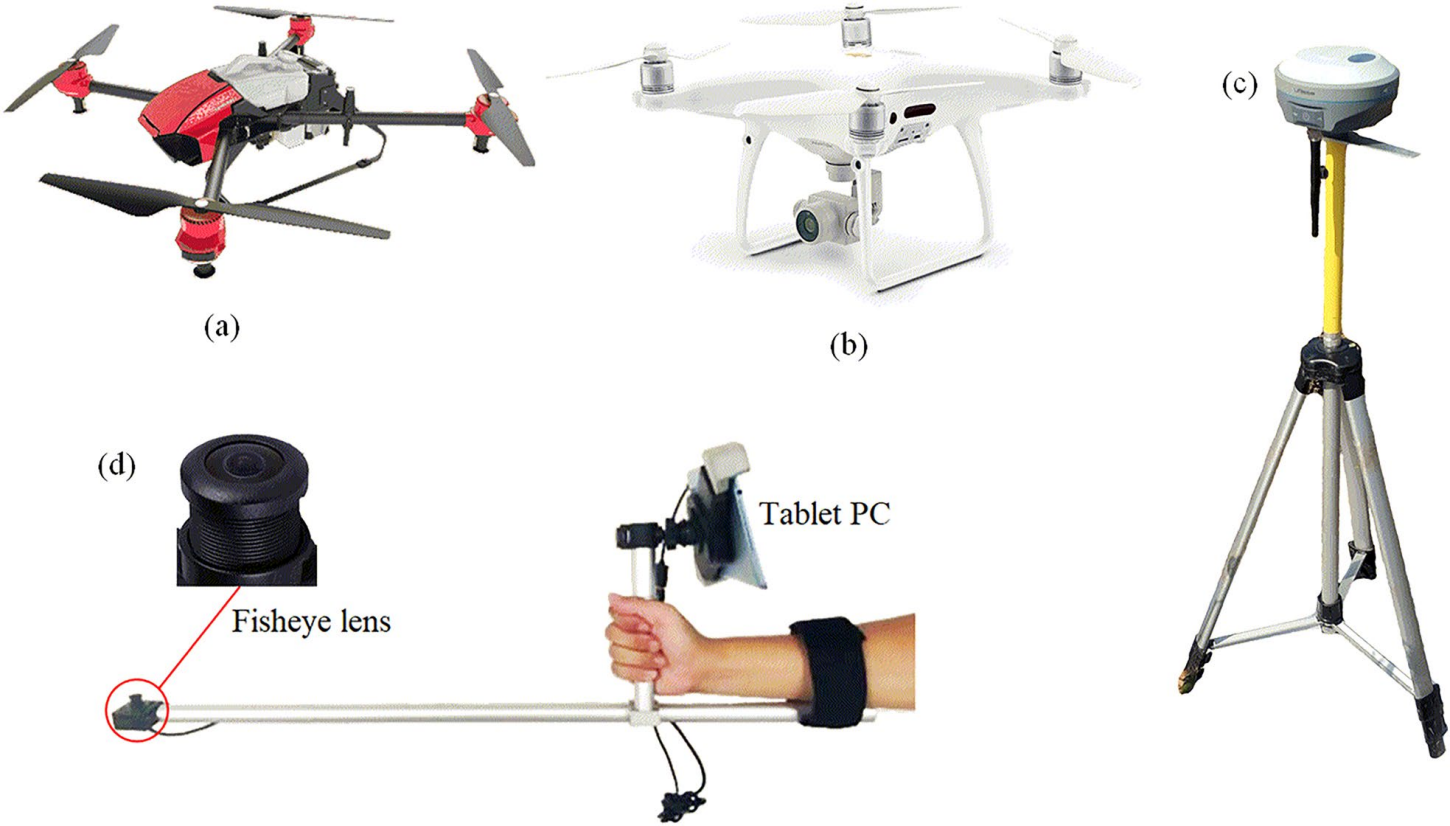
Test equipment. **a** P30 2018 UA V actual diagram. **b** A single-lens UA V for acquiring images. **c** RTK equipment. **d** LAI meter

To make the LAI of the experimental cotton field show obvious changes in a short period of time, a plant protection UAV (P30 2018, Guangzhou Jifei Technology Co., Ltd., China) was used to spray a cotton defoliant on the entire cotton field before the start of the experiment. After spraying the cotton defoliant, the cotton leaves gradually began to fall off, and the time for bolls to bloom and spit out was shortened. The flight parameters and spraying parameters of the plant protection UAV are shown in Table 1. Figure 2 shows the UAV being used to spray cotton defoliant, and Fig. 3a shows the UAV used for the spraying operation. The experimental field where the spraying operation was completed is shown in Fig. 1b.

#### Measurement of canopy height and LAI of cotton in the field

**Fig. 4:**
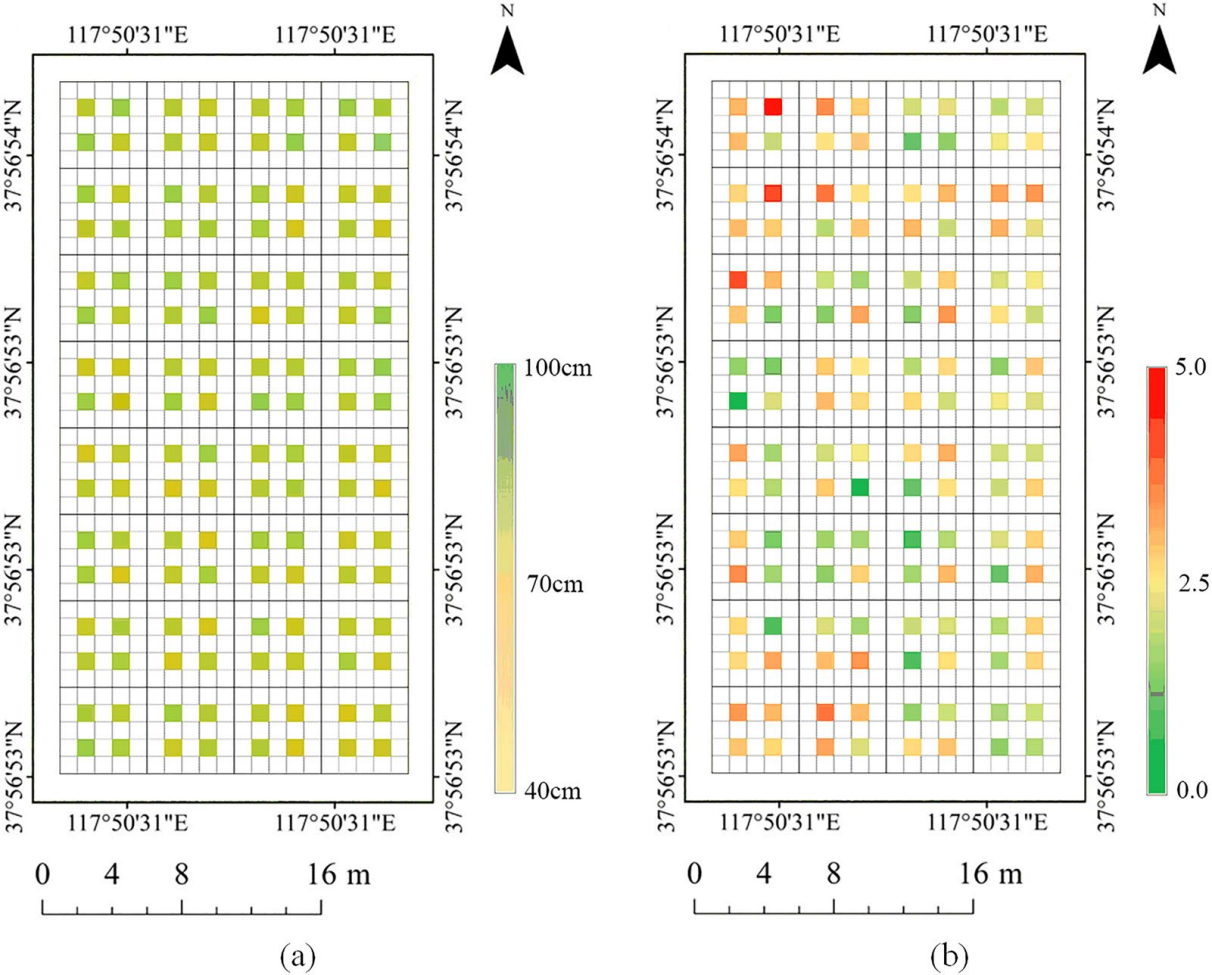
Field measurement results. **a** The actual measured height of the highest cotton in the sampling site. **b** LAI measurements on the third day after application

From the second day after the cotton defoliant was sprayed, the LAI of the test area was uniformly sampled and measured for 10 consecutive days, and the RGB image of the test area was acquired by a UAV equipped with a digital camera. The field LAI was measured using a LAI instrument (Chengdu University of Science and Technology, China); see Fig. 3d. The LAI meter is a handheld device with a fish-eye lens at the front end. The image taken by the fish-eye lens was imported into the software for analysis to obtain the LAI of the measuring point [45]. In the whole test plot, 128 small areas of 1$\times$1 m were uniformly selected to measure LAI and the highest plant height in the small area (shown in Fig. 1c, d. RTK equipment (UBase, Hi - Target Navigation technology Co., China) was used to accurately record the sampling locations, and the horizontal and vertical errors of the RTK equipment were within 1 cm and 2 cm, respectively. The RTK equipment is shown in Fig. 3c, and the distribution of measured locations is shown in Fig. 1c, d. Since the cotton at this time was already in the mature stage and the height does not change any more, the canopy height was only measured once, and the highest plant in the sampling area was selected for measurement. The measurement result of plant height is shown in Fig. 4a, and the measurement result of the LAI on the third day after application is shown in Fig. 4b.

#### Collection of UAV-based canopy RGB image

**Fig. 5:**
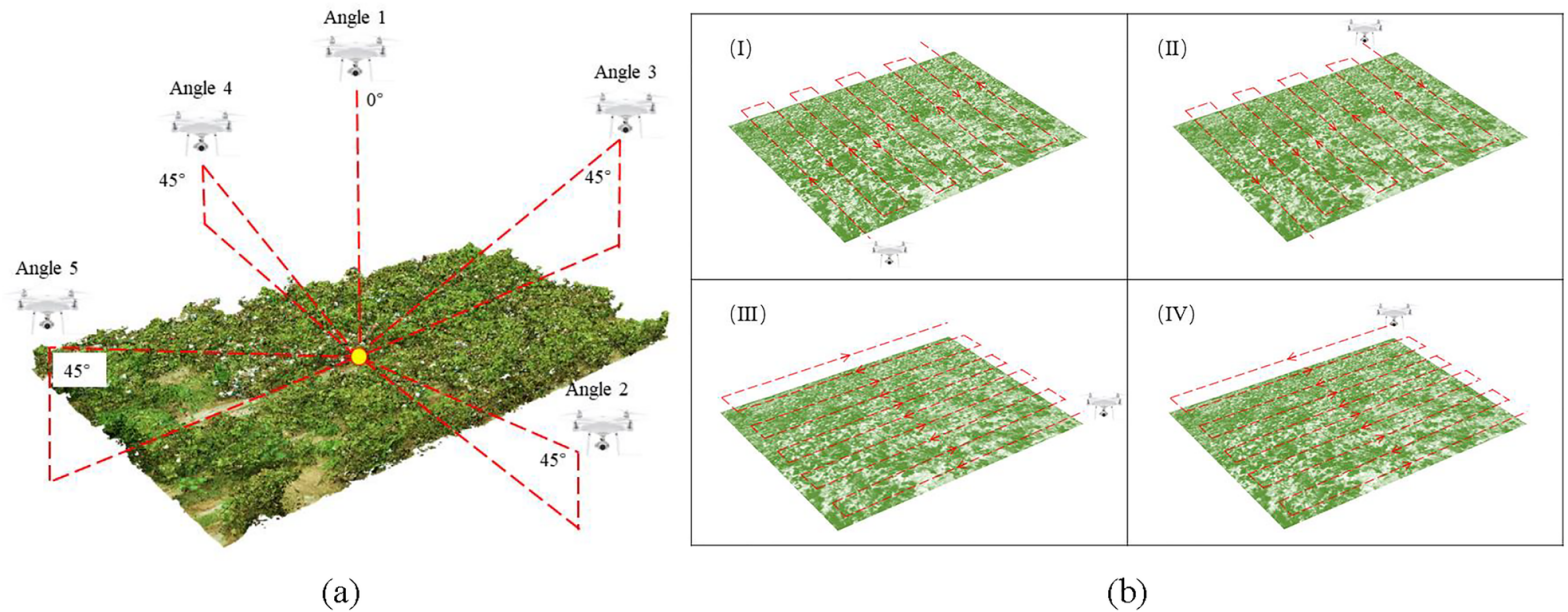
The location of the photo taken by the UAV. **a** Five different acquisition image angles. **b** Flight path of the UAV to collect images

The UAV used in the test was DJI Phantom 4 pro V2.0 (Shenzhen DJI Innovation Technology Co., Ltd., China), which is a low-cost consumer-grade quadrotor UAV equipped with a 1-inch 20 million pixel image sensor, a system composed of digital cameras (shown in Fig. 3b. Before acquiring UAV images for the first time, 9 ground control points were fixed on the test plot to perform postcalibration of UAV images. The layout of the ground control points is shown in Fig. 1c. The coordinate information of the ground control point was obtained by RTK equipment, as shown in Fig. 1e. Alizure (Shenzhen Zhuke Innovation Technology Co., Ltd., China) software was used to plan the flight parameters of the UAV. The flight altitude was 10 meters, the flight speed was 1.5 m/s, and the image forward overlap rate and side overlap rate were both set to 80$\%$. The flight time was selected at noon on a sunny day (10:00-13:00) to avoid the influence of light on the test results. To obtain high-precision results, the five-way flight mode in Alizure software was used to simulate the effect of the five-way lens. The five-way lens can simultaneously acquire images from 5 different angles every time it shoots [see Additional file 2], which is very efficient, but the price of the five-way lens is high. Therefore, we realized the effect of five-way lens based on single-lens UAV flying through multiple routes and different lens tilt angles. When acquiring images every day, the UAV performs five flight tasks. The tilt angle of the lens was set to 90$^\circ$ in the first flight and 45$^\circ$ in the remaining four flight processes, as shown in Fig. 5a. When the lens tilt angle is 90$^\circ$ (Angle 1), the flight path was any of the routes in Fig. 5b. Tilt Angle 2 in Fig. 5a corresponds to airline (III) in Fig. 5b, tilt Angle 3 in Fig. 5a corresponds to airline (II) in Fig. 5b, tilt Angle 4 in Fig. 5a corresponds to airline (IV) in Fig. 5b, tilt Angle 5 in Fig. 5a corresponds to airline (I) in Fig. 5b. After all missions were completed, approximately 950 RGB images with a spatial resolution of 0.29 cm were obtained.

### Generation of point cloud, crop surface model and orthophoto mosaice

**Fig. 6:**
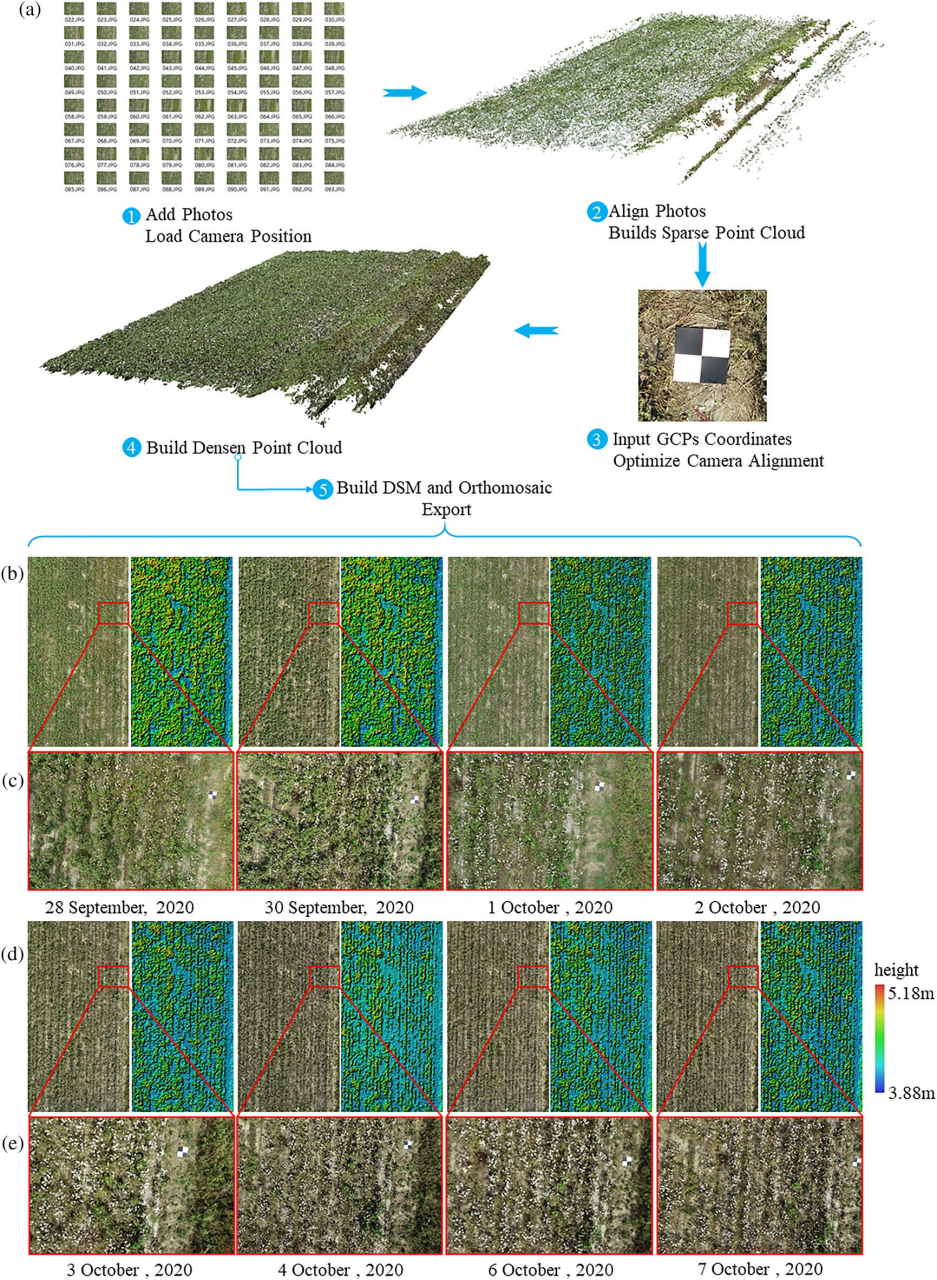
Agisoft Metashape processing workflow and exporting Orthomosaic and DSM. **a** Five processing steps in Agisoft Metashape. **b**, **d** Orthogonal mosaic with magnified views of local areas and DSM. **c**, **e** Actual cotton growth status on different dates

The point cloud of the experimental cotton field area was created using Agisoft Metashape (Agisoft LLC, St. Petersburg, Russia). The software aligns the overlapping images based on a feature point matching algorithm. The specific process of point cloud generation is shown in Fig. 6a. First, the acquired images are imported into Agisoft Metashape software for initial alignment to obtain a sparse point cloud. Then, the geographical coordinates of the ground control points were corrected, and the dense point cloud was reconstructed. Finally, Orthomosaic and DSM were constructed based on dense point clouds. In the photos obtained each time, manual screening was conducted in advance to eliminate some photos that were blurred due to special circumstances. Blurred images make it more difficult to identify key points of matching across images. In the process of generating the model, the ground control point coordinate information in the software was used to conduct manual geotagging. By identifying 9 ground markers in the model, the coordinates of ground control points were replaced with those measured by RTK equipment, and the position transformation was applied to the whole point cloud to align photos and create sparse point clouds. Ground control points were used to optimize the location and direction data of the camera to obtain more accurate processing results of geographic coordinates [39]. The generated sparse point cloud contains areas that were not needed. To save computing resources and time, only the sparse point cloud within the research area was reserved for further research. Sparse point clouds after calibrated geographic coordinates were used as input to generate dense point clouds. Since the top of the cotton canopy was sharp and small after defoliation, according to the method used by Lu et al. the recommended “mild” deep filtering was chosen to reconstruct small details to build dense point clouds [34]. Finally, based on the constructed dense point cloud, Orthomosaic and DSM of different dates were created using the default parameters in the software, as shown in Fig. 6b and Fig. 6d. Fig. 6c and e show the real growth of cotton at different times. It is obvious that there are fewer cotton leaves.

### Point cloud processing

The obtained field point cloud can be regarded as a 3D point cloud map, which is defined as a point set $S_1^{\left\{ WGS84\right\} }$

1 $$\begin{aligned} S_1^{\left\{ WGS84\right\} }=\left\{ m_i=[\alpha _i,\beta _i,\gamma _i]^T \in Q^3;i=1,...,card(S_1)\right\} \end{aligned}$$

where $\alpha _i$, $\beta _i$ and $\gamma _i$ are latitude, longitude and elevation coordinates of the World Geodetic System 1984 (WGS84), respectively. To facilitate the reading and calculation of the spatial position of the point cloud, a local Cartesian coordinate system (L-C) is redefined here. The coordinate system of the 3D point cloud was expressed from the WGS84 reference system to the L-C reference system. The position of point m was first expressed as the Earth-centered Earth-fixed coordinate system (ECEF) by using the operator f(*) to obtain the point set $S_1^{\left\{ ECEF\right\} }$. The operator f(*) is represented as the calculation procedure to transform the WGS84 coordinate system to the L-C coordinate system [29].

2 $$\begin{aligned} S_1^{\left\{ ECEF\right\} }=\left\{ m_i=[x_i,y_i,z_i]^T = f(m_i^{\left\{ WGS84\right\} }),\forall m_i^{\left\{ WGS84\right\} } \in S_1^{\left\{ WGS84\right\} } \right\} \end{aligned}$$

Each point is then represented as an L-C reference system

3 $$\begin{aligned} m_i^{\left\{ L-C\right\} }=-R_{ECEF}^{L-C} O_{L-C}^{\left\{ ECEF\right\} }+R_{ECEF}^{L-C} m_i^{\left\{ ECEF\right\} } \end{aligned}$$

where $R_{ECEF}^{L-C}$ is the rotation matrix from the ECEF reference system to the L-C reference system, and $O_{L-C}^{\left\{ ECEF\right\} }$ is the origin of the L-C reference system represented by the ECEF reference system. According to the WGS84 reference system, the location of origin $O_{L-C}^{\left\{ ECEF\right\} }$ was selected at the lowest point southwest of the test area, namely:

4 $$\begin{aligned} \begin{aligned} O_{L-C}^{\left\{ ECEF\right\} }&=\left\{ [\alpha _0,\beta _0,\gamma _0],\right. \\&\left. \alpha _0=\min (\left\{ \alpha _i:[\alpha _i,\beta _i,\gamma _i]^T \in S_1^{\left\{ WGS84\right\} } \right\} ),\right. \\&\left. \beta _0=\min (\left\{ \alpha _i:[\alpha _i,\beta _i,\gamma _i]^T \in S_1^{\left\{ WGS84\right\} } \right\} ),\right. \\&\left. \gamma _0=\min (\left\{ \alpha _i:[\alpha _i,\beta _i,\gamma _i]^T \in S_1^{\left\{ WGS84\right\} } \right\} ) \right\} \end{aligned} \end{aligned}$$

According to the definition, point $O_{L-C}^{\left\{ ECEF\right\} }$ belongs to the lower left boundary point of point set $S_1^{\left\{ ECEF\right\} }$. The rotation matrix $R_{ECEF}^{L-C}$ is defined to obtain the $x^{\left\{ L-C\right\} }$ and $y^{\left\{ L-C\right\} }$ axes tangent to the latitude and longitude of the WGS84 reference system, respectively. Here, the $x^{\left\{ L-C\right\} }$ axis is east, the $y^{\left\{ L-C\right\} }$ axis is north, and the $z^{\left\{ L-C\right\} }$ axis is opposite to the direction of the center of the Earth.

For the point cloud discussed in this paper, the origin of the local reference coordinate system was located at $[37.947351231, 117.835755425, 4.1627]^T$, and the values of matrix $R_{ECEF}^{L-C}$ and array $O_{L-C}^{\left\{ ECEF\right\} }$ are $$R_{ECEF}^{L-C}=\left[ \begin{array}{ccc} -0.8843 &{} -0.4669 &{} 0 \\ 0.2871 &{} -0.5438 &{} 0.7886 \\ -0.3682 &{} -0.6973 &{} 0.6149 \end{array} \right]$$

and

$$O_{L-C}^{\left\{ ECEF\right\} }=\left[ 2.3515,-4.4533,-3.9009 \right] ^T \cdot 10^6$$.

For ease of reading, when the superscript was not explicitly indicated below, the reference system considered is the L-C reference system by default, with $S_1^{\left\{ L-C\right\} }=S_1$. The point cloud was represented by the L-C reference system as

5 $$\begin{aligned} S_1=\left\{ m_i=[x_i,y_i,z_i]^T \in Q^3;i=1,...,card(S_1)\right\} \end{aligned}$$

where $x_i$, $y_i$ and $z_i$ are the spatial coordinates of each point in the point cloud graph.

It is a key problem to accurately extract plant height information from 3D point clouds and select points representing the top of the cotton canopy and the height of these points relative to the soil surface. To obtain the height of the point cloud in the L-C reference system, a new point set $S_2$ is established

6 $$S_{2} = \left\{ {m_{i} = [x,y,e]^{T} \in Q^{3} :\forall m = [x,y,z]^{T} \in S_{1} ,card(S_{2} ) = card(S_{1} )} \right\}$$

where, given a point $m \in S_1$, *h* is its relative height to the local ground.

By selecting sampling points and measuring the height of cotton (*h*), the ground elevation at the given point $p_i$ can be obtained. In fact, even in the plain, there may be small slopes at different sampling sites. To minimize the error, the two adjacent sampling regions were regarded as two themselves, and the terrain between the two sampling regions was modeled by defining the points of the two subsets.

7 $$\begin{aligned} M_k=\left\{ m_i=[x_i,y_i,z_i]^T \in S_2 \right\} \end{aligned}$$

and

8 $$\begin{aligned} N_k=\left\{ m_i=[x_i,y_i,z_i]^T \in S_2 \right\} \end{aligned}$$

The lowest ground elevation between the two sampling regions was determined by evaluating the centroid between $M_k$ and $N_k$, so the subset $S_k$ representing the lowest ground elevation can be simulated by plane.

9 $$\begin{aligned} \delta _j=\left\{ [x,y,z]^T \in Q^3 \sim o_{\delta _j} (x-\overline{x}_{d_j})+p_{\delta _j} (y-\overline{y}_{d_j})+q_{\delta _j} (z-\overline{z}_{d_j})=0 \right\} \end{aligned}$$

where $\overline{x}_{d_j}$,$\overline{y}_{d_j}$ and $\overline{z}_{d_j}$ are the centroid coordinates of $d_j$, and $d_j$ is the lowest subset of the centroid between $M_k$ and $N_k$. The coefficients $o_j$, $p_j$ and $q_j$ can be optimized by the following formula:

10 $$\begin{aligned} \min _{o_j,p_j,q_j \in Q} \sum _{i=1}^{card(\delta _j)}\frac{(o_j(x_i-\overline{x}_{d_j})+p_j(y_i-\overline{y}_{d_j})+q_j(z_i-\overline{z}_{d_j}))^2}{o_j^2+p_j^2+o_q^2} \end{aligned}$$

The relative height of point $m_i \subset S_k$ with respect to plane $S_k$ is

11 $$\begin{aligned} e_i=z_i+q_j^{-1}(o_j(x_i-\overline{x}_{d_j})+p_j(y_i-\overline{y}_{d_j}))-\overline{z}_{d_j},\forall m_i \subset S_k \end{aligned}$$

Finally, the 3D point cloud can be expressed as

12 $$\begin{aligned} S_3=\left\{ n=[x,y,e]^T \in Q^3 :\forall m=[x,y,z]^T \in S_k \right\} \end{aligned}$$

### Calculation of cotton canopy height

The height of the cotton canopy refers to the vertical distance between the ground and the top of the cotton canopy, such as the highest leaf in the growing period or the top of the cotton during the opening period. To estimate the plant height of crops by using aerial images taken by UAVs, the upper boundary of the crop canopy and ground altitude were usually obtained from the point cloud or DSM of the experimental area.

In this study, the UAV five-way aerial photography method was used to obtain a better image effect on the exterior of the cotton canopy, which then could construct a more accurate cotton canopy and obtain a more precise upper boundary of the canopy. The upper boundary was usually represented by a specific higher percentile in the DSM, such as the 95th or 99th percentile. However, due to the shielding between the cotton canopy in the field and the influence of weeds in the field, it is often difficult for UAV aerial photography to capture the soil information on the ground of the research area. This also makes it difficult to directly extract the ground elevation information from the reconstructed point cloud or DSM.

Currently, there are two main methods to estimate crop plant height using UAV aerial images: the point cloud method [46] and the ground reference method [47]. The point cloud method ensures that the soil on the ground within or between experimental areas can be seen by UAVs. Referring to the ground method, a digital terrain model (DTM) is generated based on aerial images of bare land before planting crops.

In the cotton growth of intensive experimental areas, the ground was shielded by the canopy, and no DTM measurement was conducted in the early stage of the test, which cannot meet the requirements of the above two methods. In this study, linear interpolation is used to generate DTM.

**Fig. 7:**
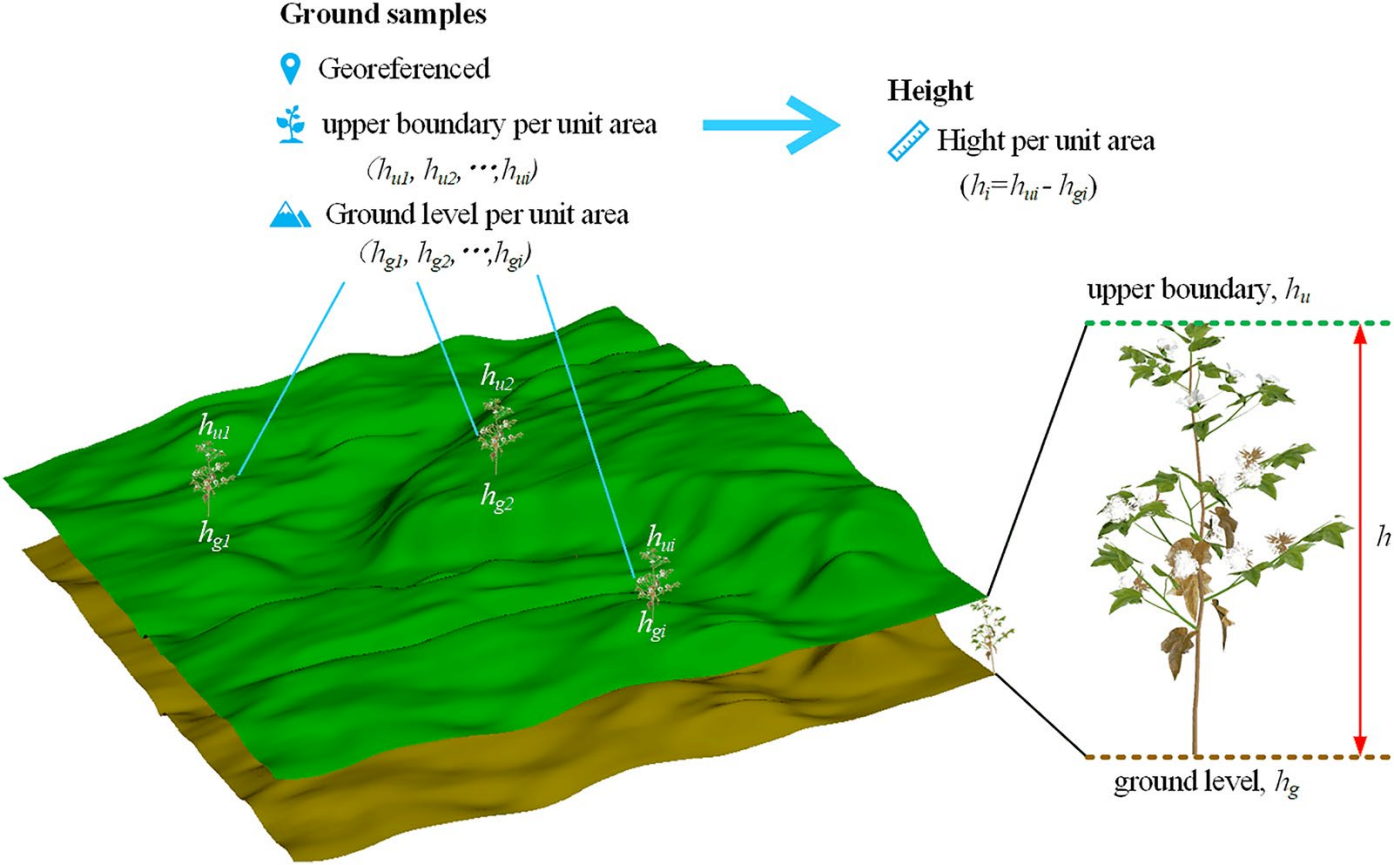
Extracts plant height *h* by subtracting ground elevation hg from the upper boundary $h_u$ of DSM

The test area was divided into 32 5$\times$5 m plots, and 4 1$\times$1 m sampling points were selected in each plot, as shown in Fig. 1 (c) and (d). According to the definition, cotton plant height (*h*) is the vertical distance between the upper boundary of the plant ($h_u$) and ground elevation ($h_g$), so we plotted an illustration figure, as shown in Fig. 7. In the process of manual measurement of cotton plant height, the highest plant was selected from four sampling points for height measurement. The ground height of the sampling point is measured by RTK equipment, the ground elevation of sampling point *i* was $h_{gi}$, and the measured plant height was $h_{ci}$. The 99th percentile of the DSM value was selected as the upper boundary in this study [47]. The upper boundary of the sampled points is denoted $P_{99i}$, and the upper boundary of the unsampled points is denoted $P_{99f}$.

Since the test plot was located in a plain area, the land leveling operation was conducted before planting cotton, the terrain was flat without undulation, and the sampling points were evenly distributed in the test area, so the ground elevation ($h_{gc}$) of the nonsampling area in the plot can be simplified by the average of the ground elevation of the four sampling points in the plot.

13 $$\begin{aligned} {h_{gf}=\frac{\sum \nolimits _{i=1}^4 P_{99i}}{4}} \end{aligned}$$

Therefore, the cotton plant height ($h_f$) of nonsampling points in the cell can be expressed as

14 $$\begin{aligned} {h_{f}=P_{99f}-h_{gf}} \end{aligned}$$

The estimation accuracy of the cotton plant height solution method was evaluated by comparing the estimated value of cotton plant height obtained by the above method with the measured value. Mean absolute error (MAE), root mean square error (RMSE) and coefficient of determination ($R^2$) were used to evaluate the accuracy of plant height estimation.

15 $$\begin{aligned}{} & {} MAE=\frac{1}{n}\sum _{i=1}^n \left| h_{ci}-h_{ei}\right| \end{aligned}$$

16 $$\begin{aligned}{} & {} RMSE=\sqrt{\frac{1}{n}\sum _{i=1}^n(h_{ci}-h_{ei})^2} \end{aligned}$$

17 $$\begin{aligned}{} & {} R^2=\frac{\sum \nolimits _{i=1}^n(h_{ei}-\overline{h})^2}{\sum \nolimits _{i=1}^n(h_{ci}-\overline{h})^2} \end{aligned}$$

where *n* is the total number of sampling points in the test field, $h_{ci}$ and $h_{ei}$ are the measured and estimated values of plant height at the *i*th sampling point, respectively, and ${\overline{h}}$ is the average value of the measured plant height at the sampling point.

### Calculation of point cloud density

**Fig. 8:**
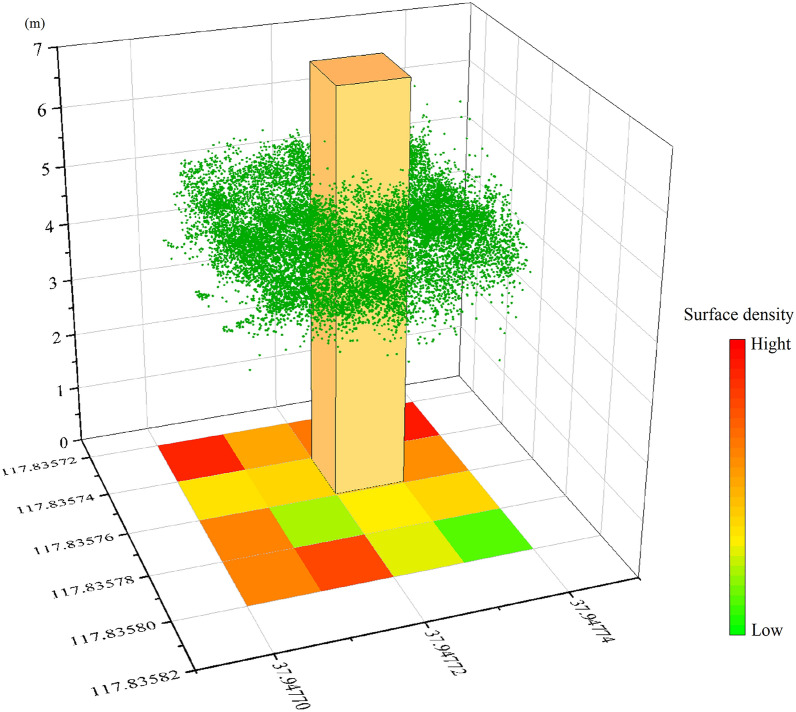
Different areas corresponding to different point cloud densities

The evaluation of cotton LAI is related to the spatial distribution of cotton leaves in the horizontal and vertical directions. Cotton in the test area was sown by machine and managed with consistent water and fertilizer. The growth pattern of cotton was basically evenly distributed in the horizontal direction, while the vertical direction showed inconsistencies in plant height due to collapse and other reasons. In this study, the vertical spatial distribution of the cotton canopy was taken as an important parameter in evaluating LAI. A single value was used to represent the complex canopy density distribution of point cloud $S_3$. To accurately analyze the point cloud density, the change in point cloud density was analyzed by defining subset $R_{u,v}$ of the point cloud within the range of $x \in [x_{min},x_{max}]$, $y \in [y_{min},y_{max}]$ and $e \in [e_{min},e_{max}]$ in point cloud $S_3$. Subset $R_{u,v}$ is in a rectangular body with equal length and width ($x=y=\epsilon$ ). The point cloud in the yellow cube in Fig. 8 is an example of subset $R_{u,v}$.

18 $$\begin{aligned}{} & {} R_{u,v}(\epsilon )=\left\{ n_i \in S_3 |(u-1) \cdot \epsilon \le x_i< u \cdot \epsilon ,(v-1)\cdot \epsilon \le e_i-e_{min}< v \cdot \epsilon \right\} \end{aligned}$$

19 $$\begin{aligned}{} & {} u \in U=\left\{ 1,2,...,\frac{x_{max}-x_{min}}{\epsilon }\right\} \end{aligned}$$

20 $$\begin{aligned}{} & {} v \in U=\left\{ 1,2,...,\frac{e_{max}-e_{min}}{\epsilon }\right\} \end{aligned}$$

By calculating the number of point clouds in subset $R_{u,v}$, all subsets $R_{u,v}$ in point gathering $S_3$ are described as a two-dimensional map

21 $$\begin{aligned} A_3=\left\{ a_{u,v}=card(R_{u,v})\cdot \epsilon ^{-2},\forall u \in U,v \in V \right\} \end{aligned}$$

When the boundary lengths *u* and *v* of subset $R_{u,v}$ are both 1, the two-dimensional map represents the density distribution of point $(S_3)_i$ in point set $S_3$ on the horizontal plane ($x-y$ plane), where $u \in [1,40],v \in [1,20]$ and $A_3$ is a two-dimensional matrix.

In the calculation of point cloud density, ground point clouds in the cell need to be segmented according to the ground elevation solved above. After segmentation, only the point clouds above the ground were retained and used as the source data for solving the point cloud density. The two-dimensional matrix $A_3$ was greatly influenced by the distribution of the cotton canopy and the $\epsilon$ value. Since the distribution of the cotton canopy could not be changed, only the $\epsilon$ value was analyzed here. Theoretically, the smaller the $\epsilon$ value is, the more detailed the two-dimensional matrix $A_3$ can reflect the heterogeneity of the density distribution of the cotton canopy point cloud. However, when the value of $\epsilon$ is too small, the number of point clouds in subset $R_{u,v}$ will be zero; that is, there are empty elements in matrix $A_3$, which will affect the effect of the density distribution map of the canopy point cloud. To better describe matrix $A_3$, $g_{x,3}(\epsilon )$ is defined to represent the ratio of the number of elements greater than 0.2 times the average density of the point cloud to the size of the total two-dimensional matrix:

22 $$\begin{aligned}{} & {} g_{x,3}(\epsilon )=[\sum _{r=1}^{(x_{max}-x_{min}) \cdot \epsilon ^{-1}} \sum _{r=1}^{(e_{max}-e_{min}) \cdot \epsilon ^{-1}} f(a_{u,v})] \times \nonumber \\{} & {} [(x_{max}-x_{min}) \cdot (e_{max}-e_{min}) \cdot \epsilon ^{-2}]^{-1} \end{aligned}$$

23 $$\begin{aligned}{} & {} f(a_{u,v})={\left\{ \begin{array}{ll} 0,a_{u,v}<0.2\rho \\ 1,a_{u,v}\ge 0.2\rho \end{array}\right. } \end{aligned}$$

where $\rho$ is the average density of the point cloud.

In combination with the definition of LAI and the planting row width of cotton, $\epsilon =1$ was chosen here.

### Construction of the LAI estimation model

There was a linear relationship between LAI and biophysical parameters of the crop. Corcoles et al. [48] showed that the linear model showed a correlation between LAI and canopy cover. The reliability of linear models in describing leaf area indices based on canopy height was demonstrated by Mathews et al. [38] Multivariate linear models can be used to describe the relationship between the defined crop canopy and LAI [13]. In this study, a multivariate linear model was used to model the LAI as follows:

24 $$LAI = \left( {\sum\limits_{{w \in G}} {t_{w} } \cdot g_{{w,3}} + j} \right) \cdot \delta _{3}^{{ - 1}}$$

where *G* is the set of selected description subset $g_{w,3}$, $t_w$ is the coefficient of description subset $g_{w,3}$, *j* is the model intercept, and $\delta$ is the cotton row spacing.

## Results

### Estimation of cotton canopy height at different times

**Fig. 9:**
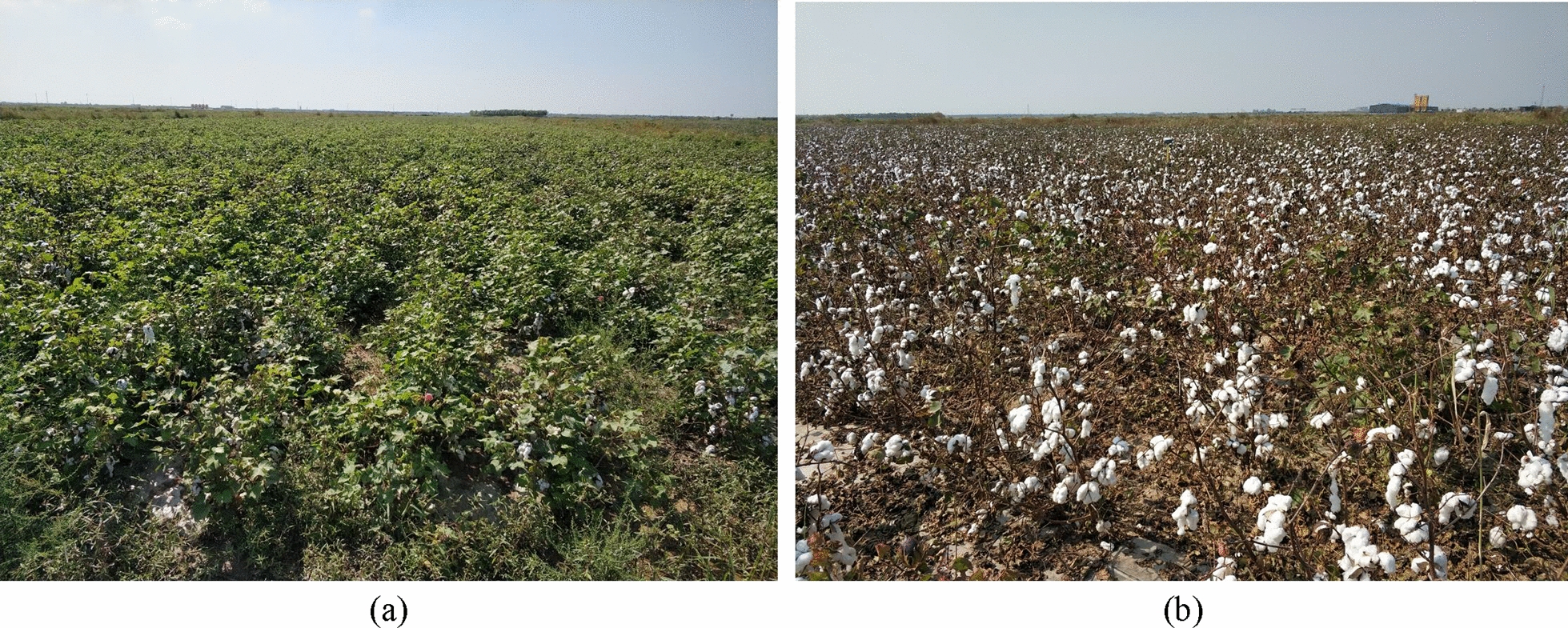
**a** Cotton after 3 days of defoliant spraying. **b** Cotton after 10 days of defoliant spraying

**Fig. 10:**
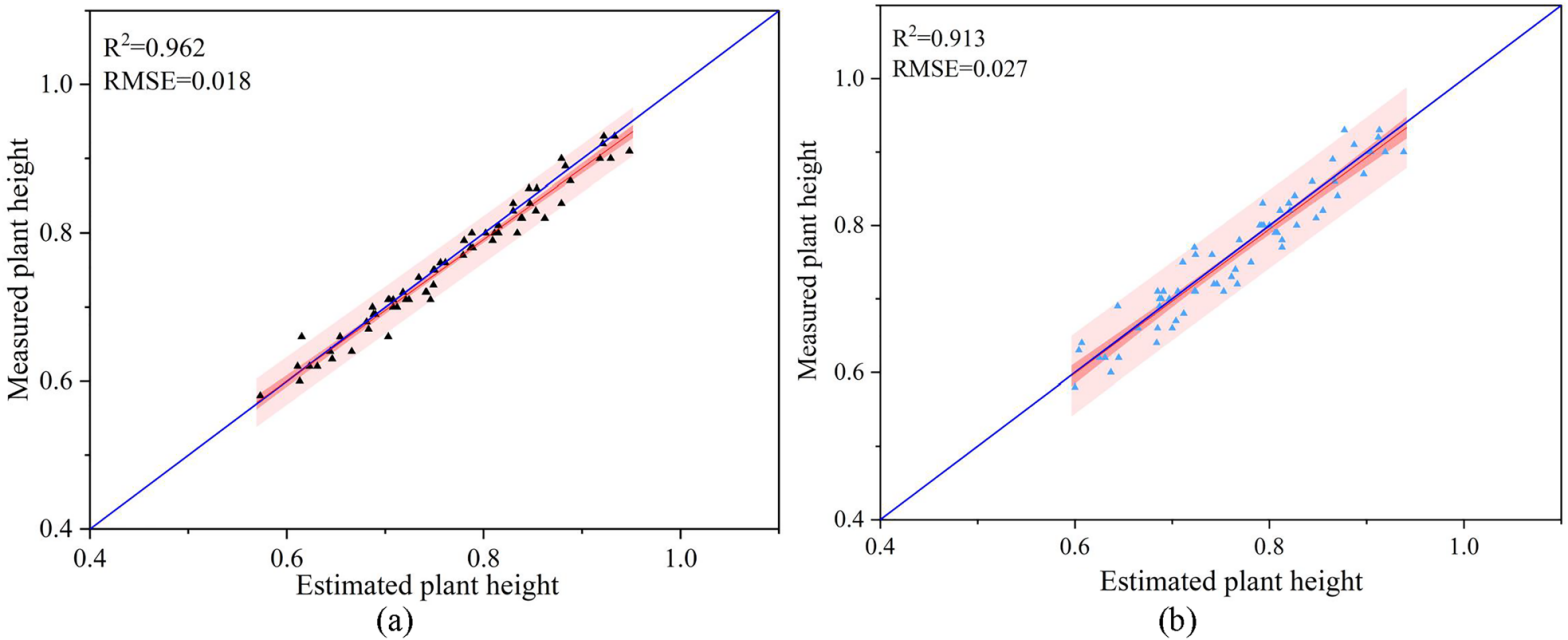
Scatter diagram of estimated plant height and actual measured plant height. **a** Estimation of plant height on October 1 (before leaf shedding). **b** Estimation of plant height on October 7 (after leaf shedding)

Three days after spraying the Cotton Defoliant, the leaves grew thickly and began to fall off, as shown in Fig. 9a. Ten days after spraying the Cotton Defoliant, the leaves basically fell off, and the cotton bolls emerged, as shown in Fig. 9b. To evaluate the predictive ability of the plant height solution method in this study, the canopy height of cotton after 3 days of defoliant spraying (before leaf shedding) and after 10 days of defoliant spraying (after leaf shedding) were estimated by using this method (shown in Eqs. (13) and (14)). Comparing the estimated height with the actual measured height (Fig. 10), the results show that the estimation effect of this method before blade shedding was due to the effect after blade shedding. The $R^2$ values were 0.962 and 0.913, and the RMSEs were 0.018 and 0.027, respectively.

### Canopy point cloud density distribution

**Fig. 11:**
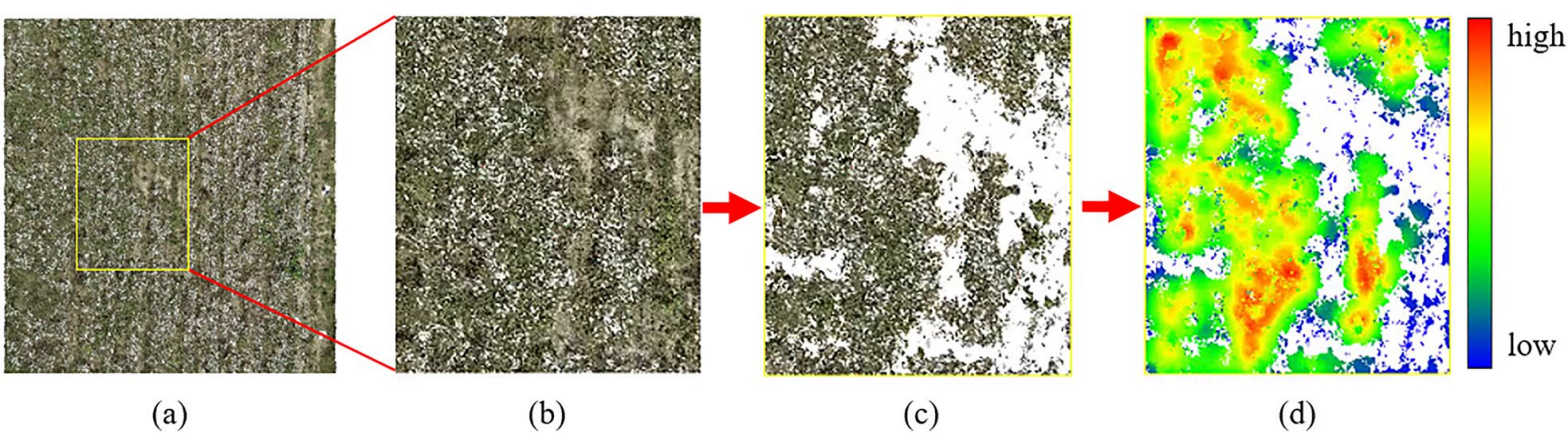
Segmentation of ground and nonground points before calculating point cloud density. **a** Randomly selected plots in the test area. **b** Enlarged view of the selected plots for analysis. **c** Point cloud of the cotton canopy after removing the ground. **d** Distribution of cotton canopy point cloud density

The distribution of point cloud density represents the growth and distribution of the cotton canopy. A large point cloud density indicates that the cotton canopy is higher or denser, which is an important factor affecting LAI in the field. To obtain the point cloud distribution of the cotton canopy, first, a plot was randomly selected in the generated dense point cloud model (Fig. 11a, b). In this study, the ground elevation of the area was obtained based on the solution of Eq. (13). The point cloud was then segmented between the ground and cotton canopy in open source software (CloudCompare v.2.11.3, CloudCompare) using the ground elevation as the reference. The segmented cotton canopy point cloud is shown in Fig. 11c. Finally, the density of the segmented cotton canopy point cloud was calculated in CloudCompare software. The calculated point cloud density distribution is shown in Fig. 11d.

### Construction of the LAI model

Field data were used to select the most reliable subset of crop descriptions by stepwise multilinear least squares optimization. A three-variable linear model for estimating the LAI was obtained by stepwise multilinear least squares optimization.

25 $$\begin{aligned} LAI = (1.37618 \cdot g_{x,3}+0.66738 \cdot d_t-0.02035 \cdot h_{ei}-0.51087)/0.76 \end{aligned}$$

where $d_t$ is the maximum diameter of the cotton canopy in the estimated area.

**Fig. 12:**
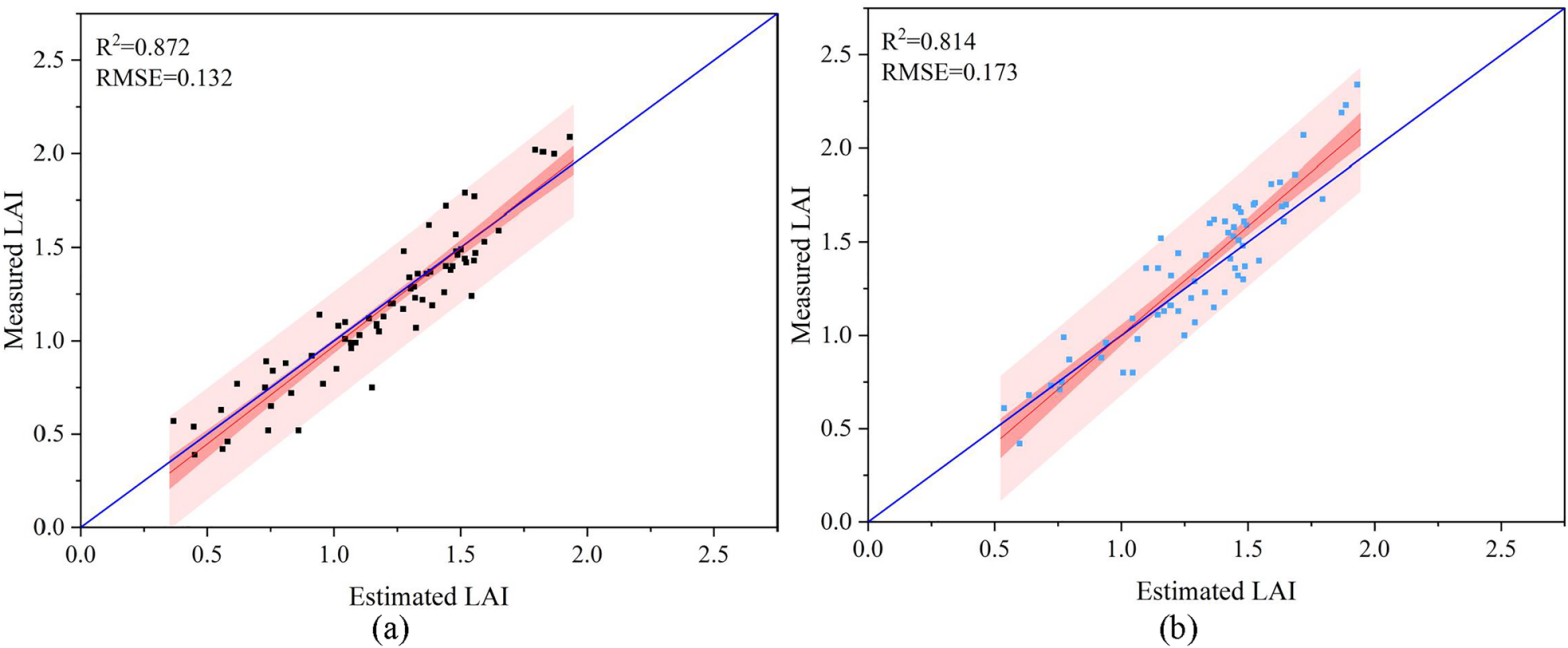
Scatter plots of estimated LAI and actual measured LAI. **a** LAI estimation on October 1 (before leaf shedding). **b** LAI estimation on October 7 (after leaf shedding)

The field LAI was estimated using Eq. (25) and compared with the actual measurements, as shown in Fig. 12. The accuracy of the estimated LAI before leaf shedding (Fig. 12a) was higher than that of the estimated LAI after leaf shedding (Fig. 12b).

## Discussion

### Accuracy difference of plant height estimation

RGB images can provide rich texture information, and RGB camera SfM technology can generate denser point cloud data, so it is suitable for producing DSMs of field crops [49]. Currently, height extraction from DSM produced by UAV aerial images is a widely used method for plant height estimation, but the accuracy still needs to be improved, especially when the ground elevation information of the experimental area is not obtained in advance. In the actual agricultural production process, there is a practical situation in which the DTM of each operation plot is difficult to obtain accurately. Since a single DSM cannot obtain accurate plant height, this study used the measured plant height of sampling points combined with DSM to reverse solve the ground elevation.

Plant height can be estimated from UAV images capturing the upper boundary of each plot (the 95th and/or 99th percentile of the DSM) and the ground elevation of each plot [50]. In this study, there was a good correlation between the plant height estimated by the UAV and that measured manually, $R^2>$0.90. Che et al. [51]. showed that both sky bottom photography and bevel photography had good consistency in estimating the plant height and LAI of maize in the field. There was a great difference in plant height estimation between the two methods, but in LAI estimation, tilting photography was better than aerial photography. However, very little research has been done in agriculture. In this study, a photographic method of a simulated five-way lens was adopted, which is a combination of sky photography and bevel photography. In the process of aerial photography, more abundant 3D cotton canopy structure information was obtained, and the constructed point cloud was more coherent and dense, especially on the side of the cotton canopy and near the root of the cotton plant. This method can provide canopy profiles of leaves and stems while ensuring accuracy in estimating plant height and LAI. The UAV image acquisition point cloud analysis method can effectively extract the phenotypic shape of cotton plants sprayed with defoliant from limited field observation data. However, at the same time, it needs special attention that the method of simulating a five-way lens needs more time and storage in data acquisition and more time to generate a dense point cloud.

The method of using the measured plant height of sampling points and DSM to reverse solve the ground elevation showed excellent estimation ability in this study even without obtaining DTM. In terms of topography, the experimental area was located in a plain area, and land leveling work was conducted before planting cotton, which satisfies the requirements for solving the ground elevation in this study. Accurate ground elevation is a prerequisite for further accurate solutions of plant height.

In Fig. 10, it can seen that the estimated plant height is more accurate before the cotton leaves fall off. The growth state and physiological characteristics of cotton will also affect the estimation accuracy of plant height, which can be summarized into three aspects. First, the flight altitude of the UAV was high, and the limited resolution of the lens limits the height information contained in each pixel point. When the top leaves of cotton fall off, the accuracy of plant height estimation decreases because the branches are thin and the canopy structure becomes sparse. Second, making DSMs using images of mailbox pixels also results in partial loss of height information [52], which makes the estimated results smaller than the actual values. In addition, if the cotton plant is swayed by natural winds, details such as end twigs may not be well reconstructed, and it is not surprising that the fidelity of the point cloud is reduced [53]. However, for the estimation of cotton plant height on flat plots, the method in this study was still applicable, and it is a good scheme for rapid monitoring of crop height in large fields.

### Differences in LAI estimates

The vertical distribution of leaf area had high genetic variability and heritability, and there was no significant difference within the same generation [54]. Leaves tend to be located in the middle or lower part of the plant in the canopy. As height increases, the distribution of leaves becomes more equally and sparsely distributed. These changes are essentially continuous in the vertical direction [54]. The total number of point clouds obtained by the five-way photography method is large and several times higher compared to traditional single vertical angle photography. A large number of relatively complete point clouds provide a relatively accurate vertical distribution of leaf area and can clearly distinguish the plant outline, but the large number of point clouds tends to lead to computational inefficiencies. The researchers found a good correlation between estimated and measured LAI using the UAV-LIDAR 3D voxel approach [55]. By filtering the appropriate voxel size to generate 3D voxels, the original shape of the point cloud can be guaranteed, the data can be compressed, and the efficiency of the algorithm can be improved. In this study, LAI was extracted at the whole canopy level, and the voxel calculation was performed on the extracted cotton canopy point cloud, which greatly reduced the computation time.

The distribution of the projection density of the point cloud reflects the growth and distribution of the cotton canopy. LAI can be estimated from the point cloud structure and density of the plant canopy [13]. In this study, the LAI was solved considering the size of the cotton canopy (canopy projected area as a percentage of the total area in the solved plot), plant height and planting row spacing. There was good agreement between the calculated LAI and the measured LAI in the period after spraying the cotton defoliant. However, the estimated correlations of cotton plants decreased after leaf shedding compared to before leaf shedding. The main reason was that after the leaves fall off, most of the cotton plants are left with only branches and cotton lint, and a small number of plants have dried leaves hanging on them. The area reflected at this time was much smaller than before the leaves fall off, which may affect the performance of SfM. Grenzdörffer et al. [52] found that for the same crop, SfM performed better in dense smooth canopies than in sparse sharp canopies. Figure 12 also shows the same result. The cotton canopy performs fully and smoothly before defoliation and has a sufficient reflective area. After defoliation, the end branches are thin. The point cloud created by the SfM method missed the information of fine branches due to the camera resolution and flight altitude. After the leaf falls off, the top and lateral branches of the plant are thin and underrepresented in the construction of DSM, which can lead to errors in the estimation of plant height and cotton crown width. These errors will eventually accumulate in the estimation of LAI, leading to the decline of $R^2$.

**Fig. 13:**
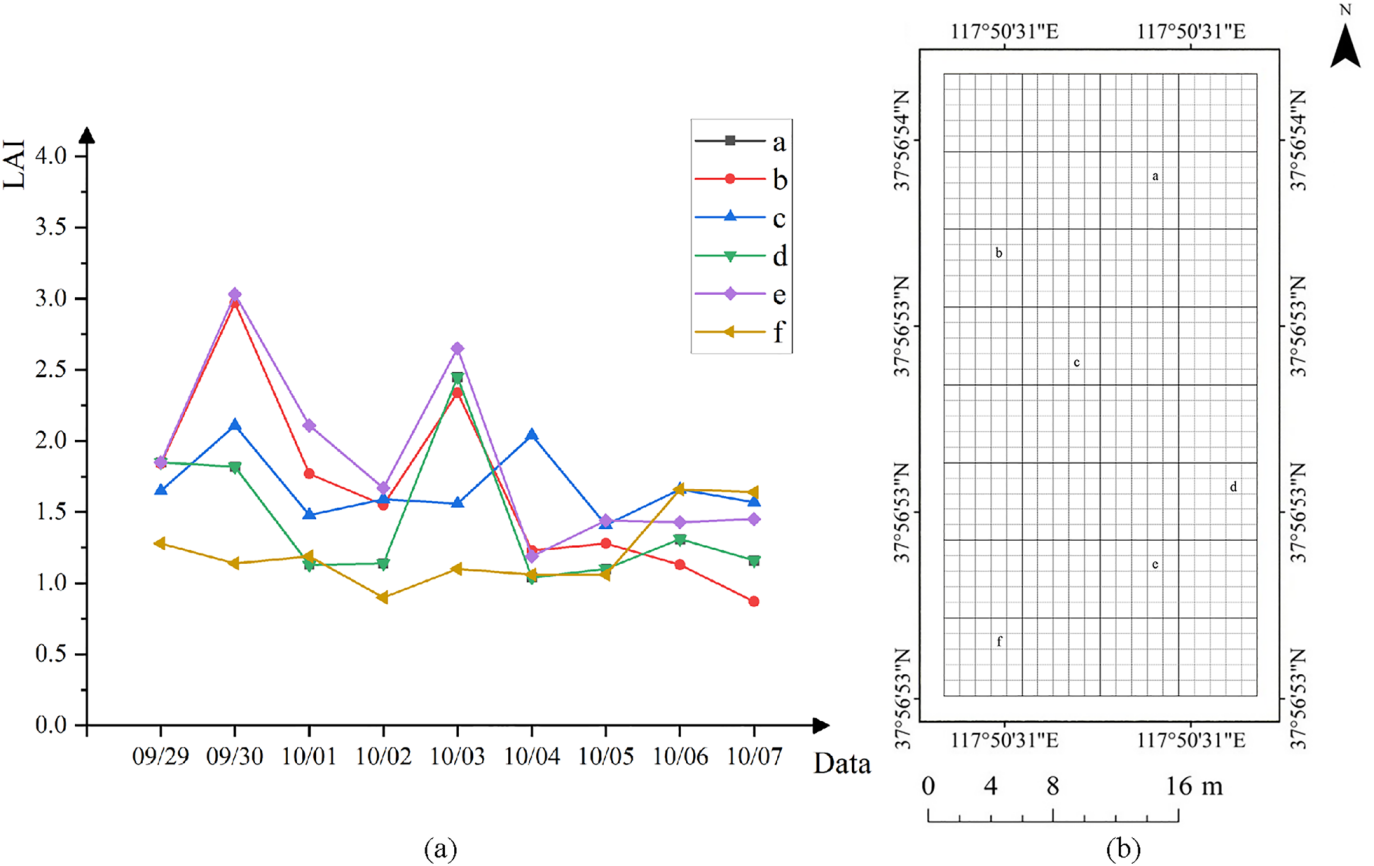
**a** LAI changes over time. **b** Sampling points for LAI measurement

Because of the use of cotton defoliants in this experiment, cotton defoliants will speed up the shedding of leaves and promote the boll opening of cotton bolls. At the later stage of the experiment, the LAI of the same measuring point changed dynamically with the shedding of cotton leaves and boll opening of cotton bolls. Figure 13 shows that during the sampling period, the LAI of cotton showed two peaks and then gradually leveled off. The main reason is that it takes some time for cotton plants to absorb cotton defoliant, and there will be an obvious defoliation effect after 2 days in general. At this time, cotton is in the early stage of flocculation, cotton plants absorb the effective ingredients of defoliant, the leaves gradually fall off the branches, and cotton bolls gradually mature flocculation. Three days after application, the volume of cotton bolls expanded rapidly after bolting, while the cotton leaves had not reached the peak of shedding; that is, the bolting speed of cotton bolls was greater than that of cotton leaves. At this time, the LAI showed the first peak, after which the leaves fell off rapidly; approximately five days after the application, most of them fell off, and the LAI was gradually reduced by the influence of cotton leaves. The cotton boll continued to produce bolls until the second peak value appeared. After reaching the peak value, seven days after treatment, cotton leaves basically fell off, and cotton bolls gradually reached the late stage of boll production. At the later stage of the experiment, LAI changed little and tended to be flat.

After cotton leaves fall off, if there is no strong wind influence, the leaves may continue to hang on the branches, which will have a certain influence on the measurement of LAI and make the measurement result slightly larger than the actual result. This study ignored the slight difference. Importantly, the constructed multitemporal point cloud can capture the trends of plant physiological parameters. Cotton bolls are not leaves, but in this study, when measuring LAI, the projected area of cotton bolls was equivalent to the spread area of leaves to verify the feasibility and accuracy of point cloud prediction of LAI.

## Conclusions

Currently, a combination of UAV aerial imagery, advanced image processing and analytical applications allows direct assessment of the phenotypic shape of cotton in the field, such as estimating the height of the cotton canopy and LAI. This technique can be a suitable method for cotton canopy height and LAI estimation, providing new opportunities to monitor the physiological traits and physical parameters of cotton in large-scale fields. As an objective, efficient, and accurate method, it can be used to replace time-consuming and laborious manual measurement. The observed data indicated that the structural changes in the cotton canopy would affect the accuracy of UAV point cloud estimation. Further studies are needed to explore this effect and the impact of agricultural environmental stroke on UAV point cloud estimation performance.

Overall, this study shows a rapid method for obtaining the plant height and LAI of cotton in the field. A small single-lens UAV was used to simulate the five-way lens for aerial photography and to generate point clouds and construct a DSM based on RGB images acquired from aerial photography in the field environment. The performance of the proposed method for estimating plant height and LAI was evaluated, and the results were satisfactory. For estimating plant height, $R^2$ and RMSE were 0.962 and 0.913 and 0.018 and 0.027, respectively. for estimating LAI, $R^2$ and RMSE were 0.872, 0.814 and 0.132, 0.173, respectively. The results demonstrated the potential of fusing manually measured plant height data and UAV aerial image data for estimating ground elevation and the potential of point clouds constructed from UAV images for estimating the LAI of cotton in the field. In future research, low-cost UAV systems can continue to be used effectively to monitor other crop growth parameters, such as aboveground biomass.

## Supplementary Information

**Additional file 1.** Spraying cotton defoliants by agricultural UAVs has become the main operation mode of mechanical cotton picking in China. (a) Indentation and cotton boll shedding formed by tractor sprayed defoliant. (b) UAV spraying defoliant does not harm crops. (c) High concentrations of Cotton Defoliant were sprayed by UAV and caused by hanging branches of coke leaves.

**Additional file 2.** A real picture of a five-way lens. There are 5 lenses in different directions mounted on one camera, which greatly improves the number and efficiency of image acquisition.

## Data Availability

The datasets used and/or analyzed during the current study are available from the corresponding authors upon reasonable request.
