# Supplementary figures and images for: Estimation of cotton canopy parameters based on unmanned aerial vehicle (UAV) oblique photography
Source: Plant Methods. 2022 Dec 8;18:129. doi: 10.1186/s13007-022-00966-z (PMC9733379; doi:10.1186/s13007-022-00966-z)

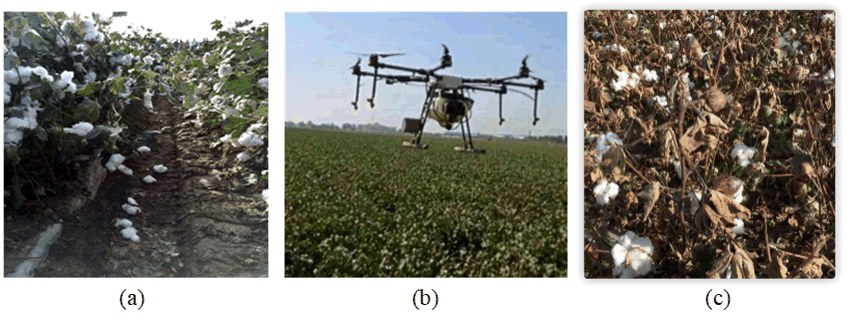

Supplement: Supplementary file 1 — Additional file 1. Spraying cotton defoliants by agricultural UAVs has become the main operation mode of mechanical cotton picking in China. (a) Indentation and cotton boll shedding formed by tractor sprayed defoliant. (b) UAV spraying defoliant does not harm crops. (c) High concentrations of Cotton Defoliant were sprayed by UAV and caused by hanging branches of coke leaves. [file 13007_2022_966_MOESM1_ESM.png]

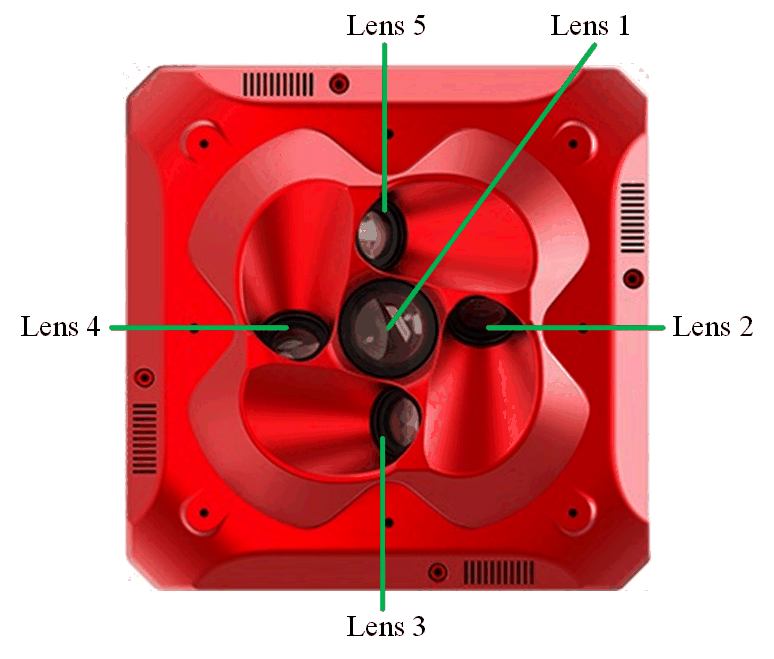

Supplement: Supplementary file 2 — Additional file 2. A real picture of a five-way lens. There are 5 lenses in different directions mounted on one camera, which greatly improves the number and efficiency of image acquisition. [file 13007_2022_966_MOESM2_ESM.png]
